# Supplementary material for: Similar but distinct: The impact of biomechanical forces and culture age on the production, cargo loading, and biological efficacy of human megakaryocytic extracellular vesicles for applications in cell and gene therapies
Source: Bioeng Transl Med. 2023 Jun 22;8(5):e10563. doi: 10.1002/btm2.10563 (PMC10486331; doi:10.1002/btm2.10563)
Supplement: Supplementary file 1 — Data S1: Supporting Information. [file BTM2-8-e10563-s001.docx]

Supplementary Materials for

**Similar but distinct: the impact of biomechanical forces and culture age on the production, cargo loading, and biological efficacy of human megakaryocytic extracellular vesicles for applications in cell and gene therapies**

Will Thompson ^1^ and Eleftherios Terry Papoutsakis ^1^

^1^ Department of Chemical and Biomolecular Engineering, University of Delaware, 590 Avenue 1743, Newark, DE 19713, USA

Corresponding author: Eleftherios Terry Papoutsakis: epaps@udel.edu; Tel: +1-302-831-8376; 590 Avenue 1743, Newark, DE 19713, USA; ORCID # 0000-0002-1077-1277

**This document includes:**

**Table S1:** Combined current and prior characterization of large MkEVs per MISEV2018 guidelines.

**Figure S1:** Experimental schematic for large MkEV isolation.

**Figure S2:** Dose-response data for control MkEVs.

**Figure S3:** Mk viability data for the syringe pump experiments.

**Figure S4:** CD54 surface expression and mean diameter for MkEVs from the shake flask experiments.

**Figure S5:** Total miRNA content for MkEVs from the shake flask experiments.

**Figure S6:** CD54 surface expression and mean diameter for MkEVs from the syringe pump experiments.

**Figure S7:** Total miRNA content for MkEVs and parent cells from the syringe pump experiments.

**Figure S8:** CD54 surface expression and mean diameter for MkEVs collected on different days.

**Figure S9:** Total and individual miRNA content in MkEVs from different days.

**Figure S10:** Bioactivity of MkEVs produced on different days.

**Table S1. Combined current and prior characterization of large MkEVs per MISEV2018 guidelines.** This table lists prior large MkEV characterization experiments for our particular culture/isolation protocol.^1-6^ Findings are categorized by the MISEV2018 criteria they meet.^7^

| **MISEV2018 Criteria** | **Assay** | **Finding** | **Source** |
| --- | --- | --- | --- |
| **Section 4a** | Counting via FC | Consistent counts; variation with biomechanical force and culture age | 1, 2, this study |
|  | Counting via NTA | Consistent counts; variation with biomechanical force and culture age | This study |
| **Section 4b** | CD41 expression | Near-universal expression; can be used as a marker for large MkEVs | 1 |
|  | CD42b expression | Moderate expression | 1 |
|  | CD62P expression | ~16% expression; suggests marginal presence of platelet-derived EVs | 1 |
|  | CD63 expression | Low expression (high expression in small MkEVs) | 2 |
|  | CD81 expression | Low expression (high expression in small MkEVs) | 2 |
|  | Total RNA profile | MkEVs enclose and transport RNA cargo; high concentration of small RNAs, lower concentration of rRNAs | 2 |
|  | miRNA sequencing | MkEV miRNA profiles differ from parent cells; 7 highly-expressed miRNAs comprise >50% of total miRNA content | 4 |
|  | Thrombopoietin contamination | Contamination is far below minimum levels required for phenotype (i.e., megakaryopoiesis of HSPCs) | 1 |
| **Section 4c** | SEM | Rough, asymmetric morphology; relatively pure samples | 2 |
|  | TEM | Lipid bilayer-enclosed vesicles; heterogeneous population; relatively pure samples | 2 |
|  | Size distribution via DLS | Consistent size distribution; distribution is distinct from that of small MkEVs | 2 |
|  | Size distribution via NTA | Consistent size distribution | 3, this study |
| **Section 5** | Basic functionality studies | Large MkEVs promote growth and megakaryocytic differentiation of HSPCs in the absence of thrombopoietin | 1, 2 |
|  | Dose-response studies | Higher doses promote more miRNA cargo uptake, megakaryocytic differentiation, and platelet production | 4, 5, this study |
|  | Uptake dependence on HSPC receptors | Uptake is largely mediated by CD54, CD11b, CD18, and CD43, but not by CD41, CD42b, or phosphatidylserine; binding and uptake occurs primarily on HSPC uropods | 2 |
|  | Uptake dependence on endocytosis | Uptake is largely dependent on lipid raft-mediated endocytosis and macropinocytosis, although membrane fusion also plays a role | 2 |
|  | Microscopy (fluorescence, SEM, TEM) of uptake | Visual evidence of MkEV uptake by HSPCs | 2 |
|  | Functionality dependence on RNA cargo | Functionality is highly dependent on RNA cargo, with notable involvement of miR-486-5p and miR-22-3p | 2, 4 |
|  | Functionality in murine models (in vivo) | MkEVs selectively localize in the bone marrow and boost platelet production | 5, 6 |

FC = flow cytometry; NTA = nanoparticle tracking analysis; SEM = scanning electron microscopy; TEM = transmission electron microscopy; DLS = dynamic light scattering.

**Figure S1**

**
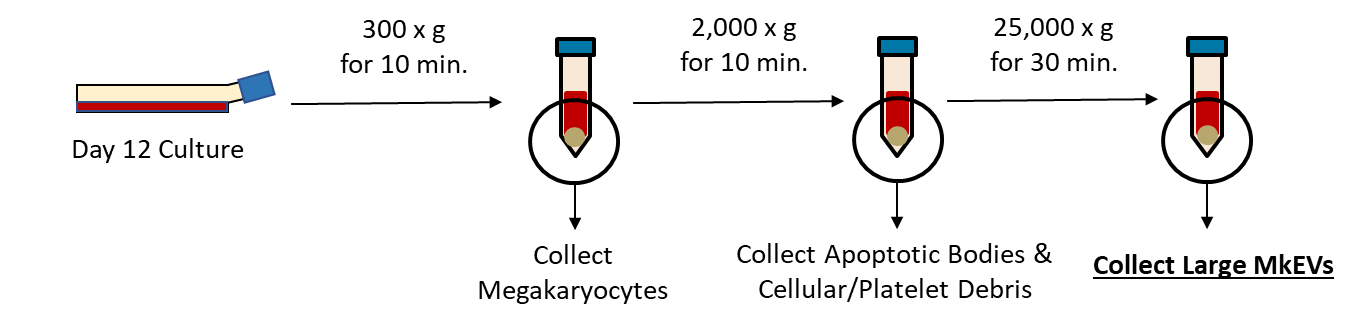
**

**Figure S1. Experimental schematic for large MkEV isolation.** Following isolation, large MkEV samples were washed three times in pure IMDM media. This protocol matches those used in our previous publications.^1-6^

**Figure S2**

**
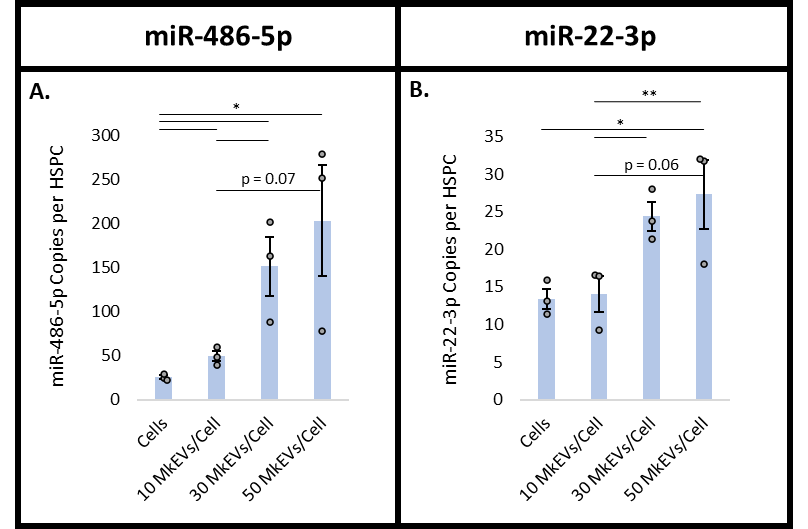
**

**Figure S2. Dose-response data for control MkEVs.** Different doses of MkEVs were co-cultured with HSPCs for 1 h. Individual miRNA levels in the HSPCs were quantified via RT-PCR. (A) Copies of miR-486-5p per HSPC following co-culture with 10, 30, or 50 MkEVs per cell. (B) Copies of miR-22-3p per HSPC following co-culture with 10, 30, or 50 MkEVs per cell. We have previously identified dose-response relationships between individual miRNA levels and megakaryocytic differentiation of HSPCs.^4^ Error bars indicate SEM of 3 biological replicates. Unpaired Student’s t-tests were performed on all data; * represents p < 0.05, ** represents p < 0.01.

**Figure S3**

**
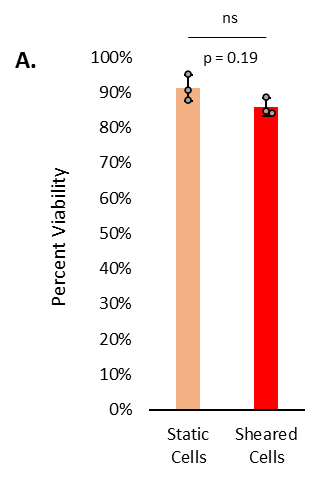
**

**Figure S3. Mk viability data for the syringe pump experiments.** (A) Viability of both control cells and cells sheared in the syringe pump was quantified using trypan blue. Error bars indicate SEM of 3 biological replicates. Paired Student’s t-test was performed on the data; ns represents non-significance.

**Figure S4**


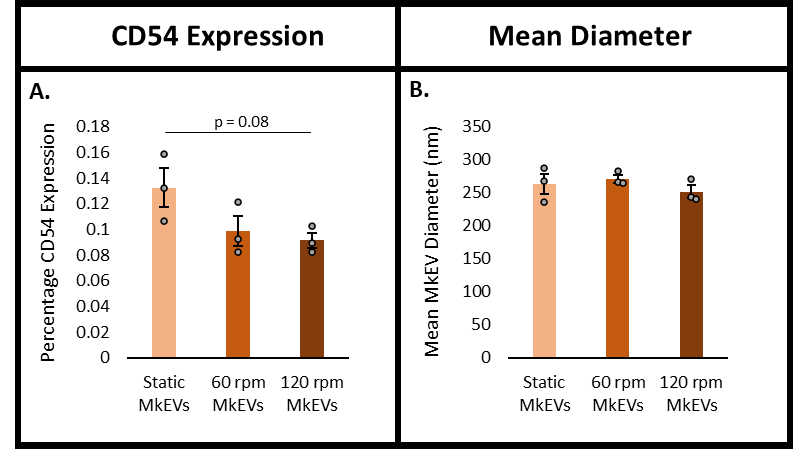


**Figure S4. CD54 surface expression and mean diameter for MkEVs from the shake flask experiments.** (A) The percentage of MkEVs (i.e., CD41^+^ particles > 200 nm in size as counted by flow cytometry) expressing CD54. (B) Mean MkEV diameter as measured by NTA. Error bars indicate SEM of 3 biological replicates. Paired Student’s t-tests were performed on all data.

**Figure S5**

**
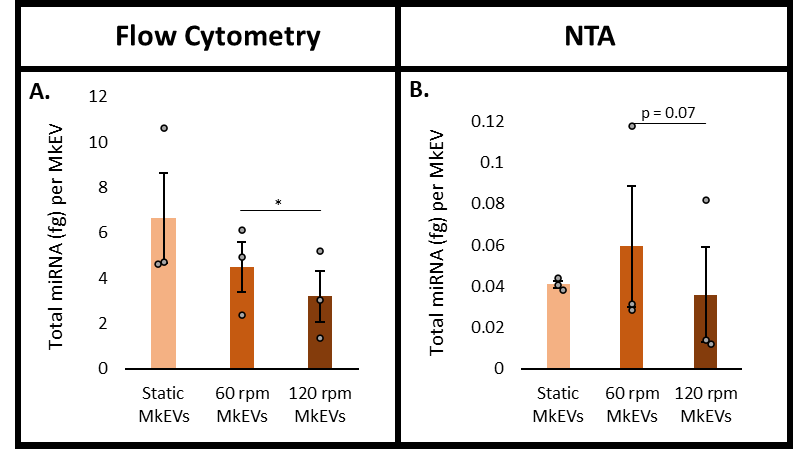
**

**Figure S5. Total miRNA content for MkEVs from the shake flask experiments.** (A) Total miRNA (in femtograms) per MkEV for flow cytometry-based MkEV counts. (B) Total miRNA (in femtograms) per MkEV for NTA-based MkEV counts. Error bars indicate SEM of 3 biological replicates. Paired Student’s t-tests were performed on all data; * represents p < 0.05.

**Figure S6**


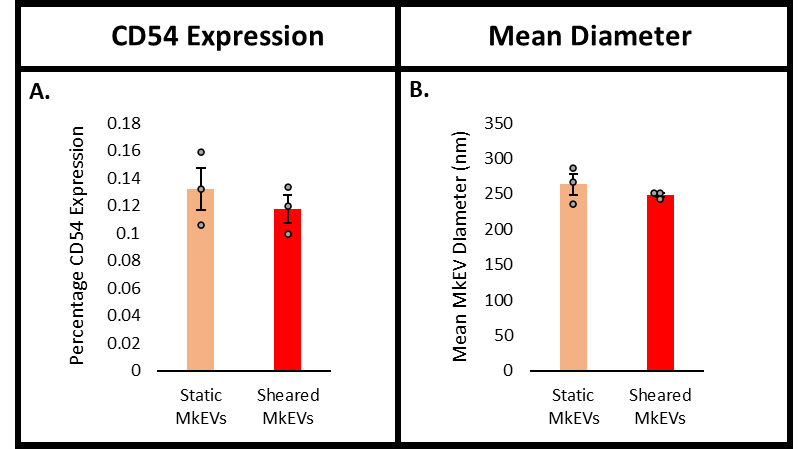


**Figure S6. CD54 surface expression and mean diameter for MkEVs from the syringe pump experiments.** (A) The percentage of MkEVs (i.e., CD41^+^ particles > 200 nm in size as counted by flow cytometry) expressing CD54. (B) Mean MkEV diameter as measured by NTA. Error bars indicate SEM of 3 biological replicates. Unpaired Student’s t-tests were performed on all data.

**Figure S7**

**
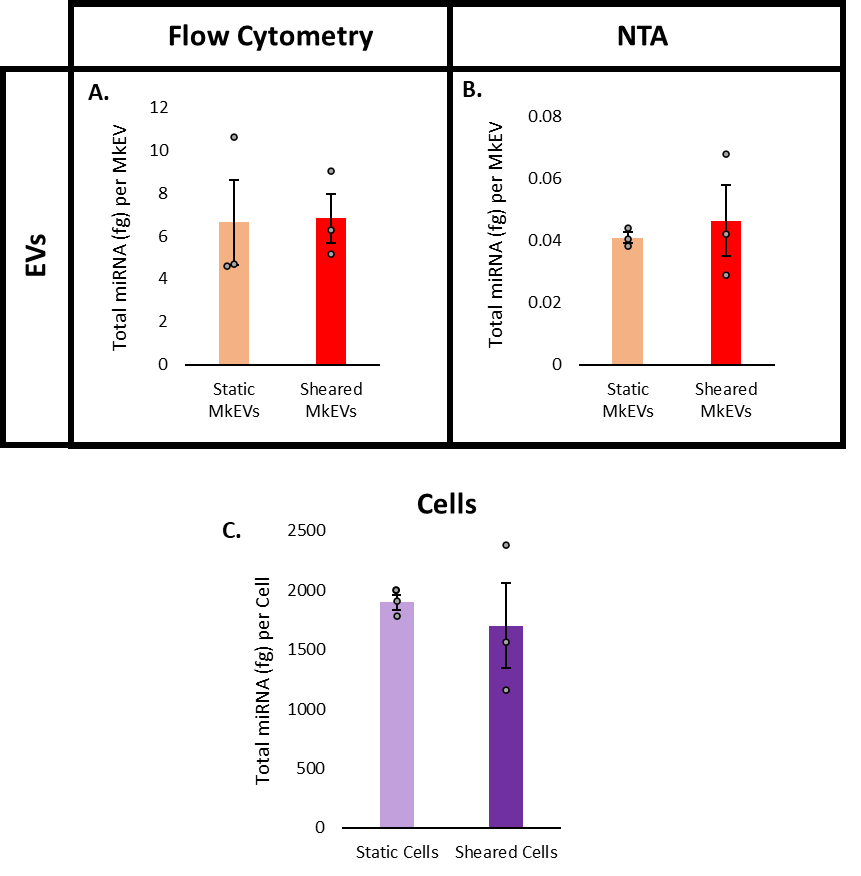
**

**Figure S7. Total miRNA content for MkEVs and parent cells from the syringe pump experiments.** (A) Total miRNA (in femtograms) per MkEV for flow cytometry-based MkEV counts. (B) Total miRNA (in femtograms) per MkEV for NTA-based MkEV counts. (C) Total miRNA per Mk following syringe pump-induced shear or control treatment. Error bars indicate SEM of 3 biological replicates. Unpaired Student’s t-tests were performed on all data.

**Figure S8**

**
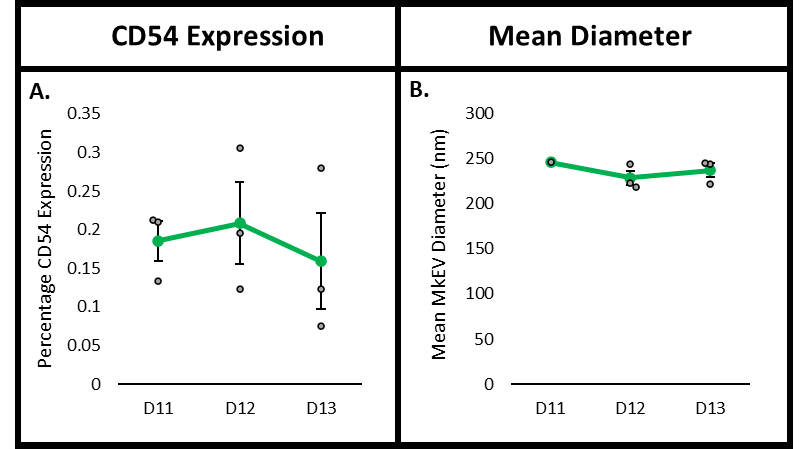
**

**Figure S8. CD54 surface expression and mean diameter for MkEVs collected on different days.** (A) The percentage of MkEVs (i.e., CD41^+^ particles > 200 nm in size as counted by flow cytometry) expressing CD54. (B) Mean MkEV diameter as measured by NTA. Error bars indicate SEM of 3 biological replicates; data points using NTA-derived D11 MkEV counts consist of 1 biological replicate each. Paired Student’s t-tests were performed on all data.

**Figure S9**

**
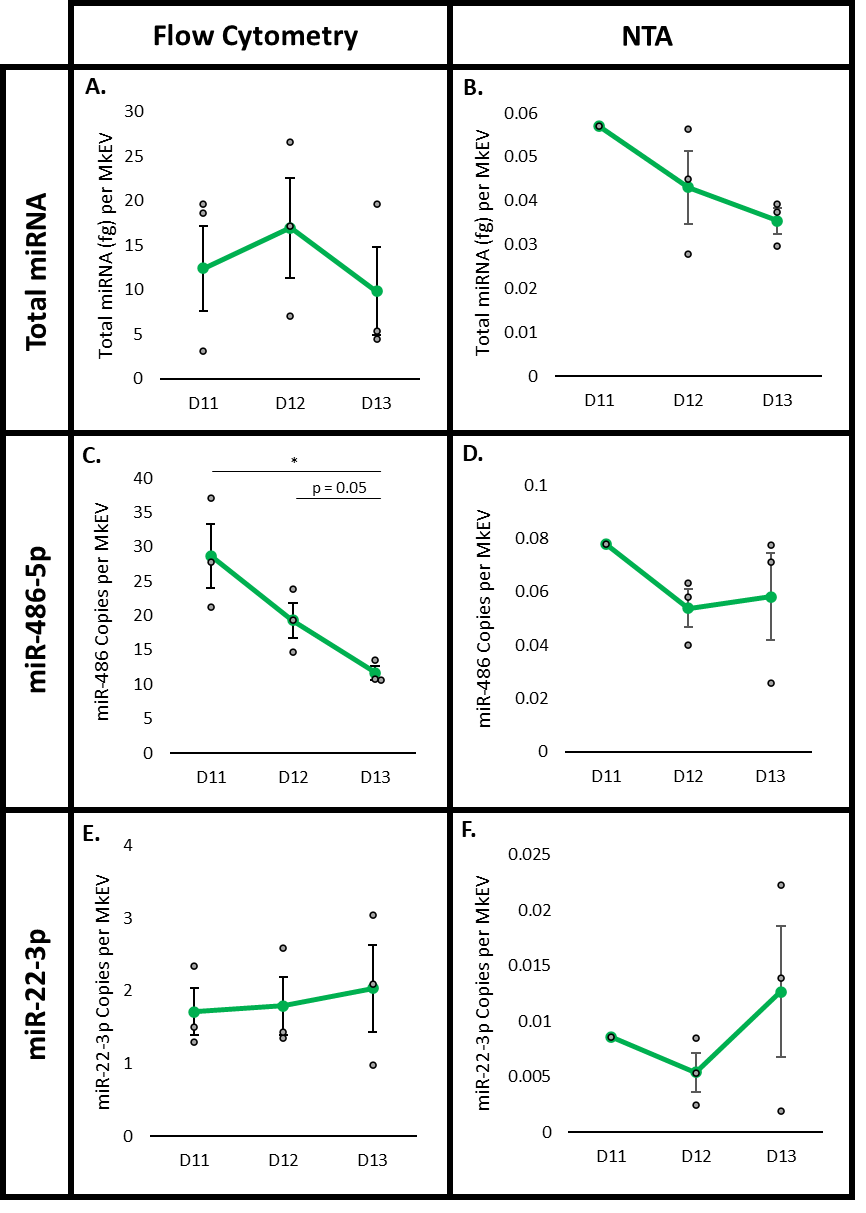
**

**Figure S9. Total and individual miRNA content in MkEVs collected on different days.** (A) Total miRNA (in femtograms) per MkEV from D11-D13 for flow cytometry-based MkEV counts. (B) Total miRNA per MkEV from D11-D13 for NTA-based MkEV counts. (C) Copies of miR-486-5p per MkEV from D11-D13 for flow cytometry-based MkEV counts. (D) Copies of miR-486-5p per MkEV from D11-D13 for NTA-based MkEV counts. (E) Copies of miR-22-3p per MkEV from D11-D13 for flow cytometry-based MkEV counts. (F) Copies of miR-22-3p per MkEV from D11-D13 for NTA-based MkEV counts. Error bars indicate SEM of 3 biological replicates; data points using NTA-derived D11 MkEV counts consist of 1 biological replicate each. Paired Student’s t-tests were performed on all data; * represents p < 0.05.

**Figure S10**

**
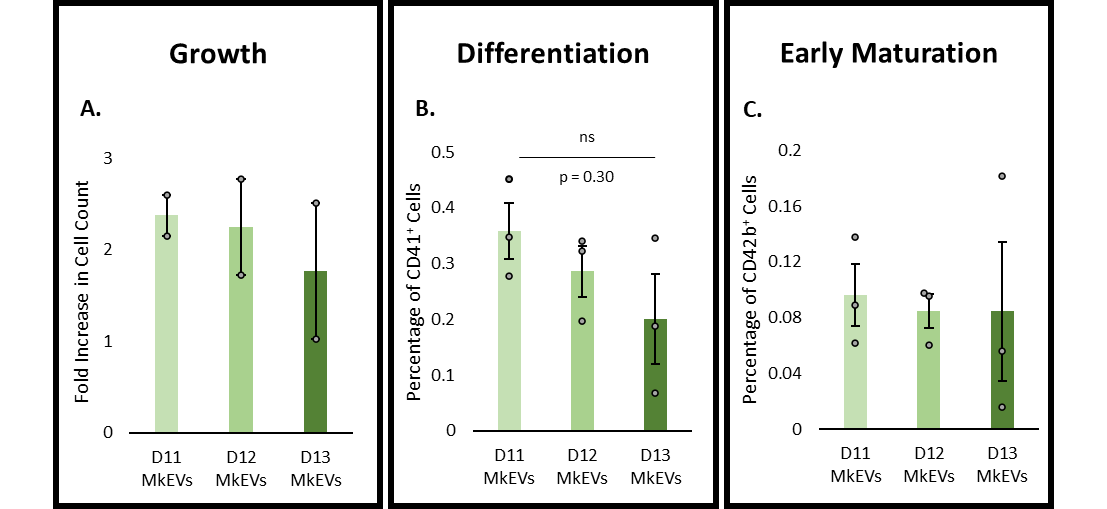
**

**Figure S10. Bioactivity of MkEVs collected on different days.** MkEVs from D11, D12, and D13 Mk cultures were co-cultured with HSPCs at a 20:1 ratio for 7 days. (A) Fold change in cell growth (relative to untreated cells) following co-culture with various MkEV samples. (B) The percentage of cells in each co-culture expressing CD41 (an Mk marker). (C) The percentage of cells in each co-culture expressing CD42b (a marker for early Mk maturation). Error bars indicate SEM of 3 biological replicates, except for (A), which shows 2 biological replicates. Paired Student’s t-tests were performed on all data; ns represents non-significance.

**References**

**1.** Jiang J, Woulfe DS, Papoutsakis ET. Shear enhances thrombopoiesis and formation of microparticles that induce megakaryocytic differentiation of stem cells. *Blood.* 2014;124(13):2094-2103. doi:10.1182/blood-2014-01-547927

**2.** Jiang J, Kao CY, Papoutsakis ET. How do megakaryocytic microparticles target and deliver cargo to alter the fate of hematopoietic stem cells? *J Control Release.* 2017;247:1-18. doi:10.1016/j.jconrel.2016.12.021

**3.** Kao C-Y, Papoutsakis ET. Engineering human megakaryocytic microparticles for targeted delivery of nucleic acids to hematopoietic stem and progenitor cells. *Science Advances.* 2018;4. doi:10.1126/sciadv.aau6762

**4.** Kao C-Y, Jiang J, Thompson W, Papoutsakis ET. miR-486-5p and miR-22-3p Enable Megakaryocytic Differentiation of Hematopoietic Stem and Progenitor Cells without Thrombopoietin. *International Journal of Molecular Sciences.* 2022;23(10). doi:10.3390/ijms23105355

**5.** Escobar C, Kao CY, Das S, Papoutsakis ET. Human megakaryocytic microparticles induce de novo platelet biogenesis in a wild-type murine model. *Blood Adv.* 2020;4(5):804-814. doi:10.1182/bloodadvances.2019000753

**6.** Das S, Thompson W, Papoutsakis ET. Native and engineered human megakaryocytic extracellular vesicles for targeted non-viral cargo delivery to blood stem cells. *bioRxiv.* 2023:2023.2004.2011.536479. doi:10.1101/2023.04.11.536479

**7.** Thery C, Witwer KW, Aikawa E, et al. Minimal information for studies of extracellular vesicles 2018 (MISEV2018): a position statement of the International Society for Extracellular Vesicles and update of the MISEV2014 guidelines. *J Extracell Vesicles.* 2018;7(1):1535750. doi:10.1080/20013078.2018.1535750
